# Protecting Children from Environmental Toxins

**DOI:** 10.1371/journal.pmed.0020061

**Published:** 2005-03-29

**Authors:** Bruce P Lanphear, Charles V Vorhees, David C Bellinger

## Abstract

Lanphear and colleagues argue that the existing requirements in the US for toxicity testing and regulation of pesticides and industrial chemicals are inadequate to safeguard children

## Teaser

Toxicity testing of pesticides and industrial chemicals is a crucial step

Epidemics of overt toxicity following widespread environmental contamination from commercial toxins heralded the discovery of children's enhanced vulnerability to lead, methyl mercury, polychlorinated biphenyls (PCBs), and tobacco [1,2,3,4,5] (Box 1). Over the past three decades, researchers have found that remarkably low-level exposures to these toxins are linked with less overt symptoms of toxicity—intellectual impairments, behavioral problems, spontaneous abortions, or preterm births [6,7,8,9,10,11,12,13,14, 15,16,17,18,19,20,21,22,23,24,25,26,27, 28,29,30,31,32,33,34,35,36,37,38,39,40]. Moreover, there is emerging evidence that decrements in intellectual abilities and low birth weight linked with lead or tobacco are, for a given increment of exposure, greater at lower levels than those found at higher levels [10,41,42,43].

**Box 1. Poisoning following Widespread Environmental Contamination from Commercial Toxins**

- **Lead:** One hundred years ago, an epidemic of lead poisoning was described among children who ingested leaded house paint [2,3]. The children developed anemia, encephalopathy, paralysis, and blindness.
- **Methyl Mercury:** In the 1950s, in the Japanese fishing village Minamata Bay, which was contaminated with methyl mercury, children developed cerebral palsy, limb defects, and mental retardation [4].
- **PCBs:** In Taiwan and Japan during the 1960s and 1970s, the ingestion of PCB-contaminated rice bran oil by pregnant women led to fetal wasting and cola-colored, dull, apathetic children [5].
- **Tobacco:** During the past century, widespread tobacco use has led to an epidemic of undersized, premature babies and children with repeated bouts of wheezing or asthma [6,7,8,9,10].

The consequences of exposure to many other chemicals or mixtures of chemicals, such as insecticides—chemicals oftentimes specifically designed to be toxic—are largely unknown [33,34,35,44]. Many of these chemicals or their metabolites are routinely found in the blood and body fluids of pregnant women and children [45].

## Children's Vulnerability to Environmental Toxins

The developing fetus and young child is particularly vulnerable to certain environmental toxins [46,47,48,49,50]. Critical neurodevelopmental processes occur in the human central nervous system during fetal development and in the first three years of life. These processes include cortical functional differentiation, synaptogenesis, myelination, and programmed apoptosis [46].

Children's exposure to environmental toxins is insidious. Environmental toxins covertly enter a child's body transplacentally during fetal development or by direct ingestion of house dust, soil, and breastmilk and other dietary sources during early childhood [51,52,53,54,55,56]. Our ability to directly measure the actual levels of environmental chemicals in human tissues and body fluids using biologic markers (biomarkers) enables scientists to more effectively link exposures to environmental toxins with disability or disease [57].

Despite our increased knowledge of the toxicity of environmental chemicals, testing for developmental neurotoxicity (DNT) and reproductive toxicity is rarely done. DNT testing uses animal experiments to provide information on the potential functional and morphologic toxicity to the fetal nervous system that results from the mother's exposure to toxins during pregnancy and lactation. Paradoxically, DNT testing of a chemical is seldom requested, and then typically requested only if there is pre-existing evidence that it is neurotoxic.

## The Prevalence of Diseases and Disabilities Linked to Environmental Toxins

Based on parental reports, one in six United States children has one or more developmental disabilities, from a subtle learning disability to overt behavioral or emotional disorders [58]. Exposures to environmental toxins have been linked with higher rates of mental retardation, intellectual impairment, and behavioral problems, such as conduct disorder and attention deficit hyperactivity disorder [16,17,18, 19,20,21,22,23,24,25,26,27,30,31,36,37, 38,39,40,41,42,43,59,60,61].

One in ten US babies is born preterm and about 5% have low birth weight [62,63]. Preterm birth, defined as birth at less than 37 weeks of gestation, is a major determinant of infant mortality and morbidity throughout childhood [62,63,64]. Exposures to environmental toxins such as lead, tobacco smoke, and DDT have been linked with an increased risk for spontaneous abortion, low birth weight, or preterm birth [6,9,10,13,14,15,28,32,65,66]. The rate of occurrence for many of these diseases or disabilities has been rising, as has treatment for attention deficit hyperactivity disorder and depression in children [62,63,67,68,69,70].

Multiple risk factors, including both genetic and environmental influences, interact in complex and often unknown ways to cause disease and disability in children. But efforts can be undertaken to prevent or reduce environmental exposures linked to disease without full elucidation of the underlying mechanism [71]. Thus, conducting some sort of test to identify pesticides and industrial chemicals that could cause reproductive or neurobehavioral toxicity before the chemical reaches widespread use is essential to protect pregnant women and children.

## Origin and Evolution of DNT Tests

The process for testing potential developmental neurotoxins in laboratory animals evolved out of a series of tragic epidemics. Widespread use of the drug thalidomide during the 1950s led to an epidemic of phocomelia, an absence or deformity of limbs and other congenital defects in children exposed in utero to the drug [72]. Subsequently, in 1965, the Food and Drug Administration (FDA) developed the Teratology Guidelines. Because thalidomide induced gross defects in rabbits but not in rats, these guidelines called for toxicity tests in two species. Moreover, these guidelines focused on gross abnormalities; they did not require testing for behavioral or DNT.

Following the outbreak of methyl mercury poisoning in Minamata Bay (Box 1), Japan and the United Kingdom added behavioral (DNT) guidelines to their teratology requirements in 1974 and 1975, respectively [73]. In 1978, the Collaborative Behavioral Teratology Study (CBTS) was conceived to standardize and evaluate methods for DNT testing in the US [74]. The final report was issued in 1985, and shortly thereafter, Dr. Donald Kennedy, who was then Commissioner of the FDA, supported the adoption of the CBTS recommendations. But the FDA failed to implement these recommendations after Kennedy's departure.

Children's exposure to environmental toxins is insidious

In 1990, the US Environmental Protection Agency (EPA) identified nine developmental neurobehavioral teratogens for both humans and animals (lead, PCBs, methyl mercury, cocaine, alcohol, phenytoin, heroin, methadone, and ionizing radiation) and developed rules for DNT testing in laboratory animals [49,50]. By 1991, the Developmental Neurotoxicity Test Guidelines (OPPTS 870.6300) had been established for use when submitting chemical data to the EPA [49]. In 1993, the National Research Council recommended that DNT data be included in the EPA's evaluations of pesticides, which include classes of chemicals specifically designed to be toxic [44].

## The Precarious US Framework for Protecting Children

Despite numerous attempts to upgrade the regulatory system, such as the CBTS, the framework to protect children from environmental toxins is precarious. Under current regulations, manufacturers of commercial chemicals (excluding pesticides) are not required to supply any toxicity data before selling their products. Nor are pesticide manufacturers obligated to supply basic premarket toxicity and exposure data necessary to ensure that children will be protected from exposure and potential harm from use of those pesticides. Indeed, the vast majority of chemicals have not been tested for DNT. The most basic toxicity tests in animals are lacking for 75% of the 3,000 highest production volume chemicals—chemicals for which annual production exceeds 1 million pounds per year [49,75,76,77]. The US EPA has entered into an agreement with the American Chemistry Council, the chemical manufacturer's trade association, to provide basic toxicity screening tests for the high-production-volume chemicals by 2005 (http://www.epa.gov/chemrtk/volchall.htm), but this is voluntary.

For new pesticides intended for use on food crops—one of the areas in which regulations are most stringent—regulations require only that DNT testing be evaluated for substances already known or suspected of being toxins. Further, neurotoxicity testing need be conducted only in adult animals. The EPA acknowledges that over 140 registered pesticides are neurotoxic (i.e., specifically designed to act against pests by interfering with neurotransmitters or other processes shared by mammals and insects), but the EPA has received DNT testing using validated protocols for only nine pesticides [49,75,76,77].

There is no general requirement that pesticides or other chemicals be tested for potential DNT prior to their registration and use [49]. For pesticides—which undergo more premarket testing than other chemicals—the EPA has relied on a tiered system of toxicity testing. The assumption underlying this system is that positive findings on earlier, more basic tests of neurotoxicity in adult animals will “trigger” the EPA to request more extensive testing by manufacturers, including tests in immature animals. Unfortunately, this tiered process has failed to result in appropriate DNT testing. In 1998, an internal EPA Toxicology Working Group concluded that these triggers may not be sufficient to identify all chemicals that have the potential to produce DNT [75]. Moreover, this tiered system discourages industry from conducting testing in immature animals because the findings could necessitate further costly testing and hinder a chemical from reaching the market.

## The European Framework: “REACH”

**Figure 1:**
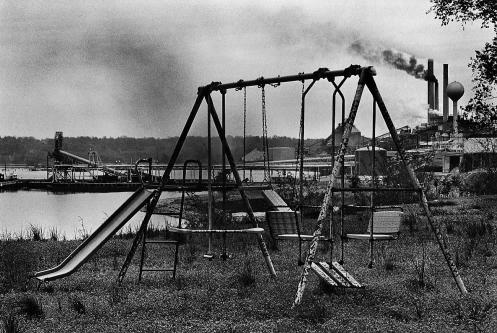
Flow Chart Summarizing REACH (Registration, Evaluation, and Authorization of Chemicals)—the European Commission's Regulatory Framework for Chemicals (Illustration by Sapna Khandwala, Public Library of Science, adapted from [86])

In 2001, the European Commission affirmed that the European Union's legislative framework did not provide adequate information about the adverse effects of chemicals on human health, and that when hazards were identified the regulatory agencies were slow to assess risks and to introduce measures to reduce those risks [78]. Indeed, chemical manufacturers are not required to “prove” that a chemical is safe before marketing it. The European Commission proposed a new regulatory framework for chemicals, REACH (Registration, Evaluation, and Authorization of Chemicals) [78,79] (Figure 1).

Under REACH, chemical manufacturers would have to assume a much greater burden for showing the lack of harm from use of their products. Specifically, REACH would require both European and non-European manufacturers doing business in Europe to submit more extensive toxicity data for about 30,000 chemicals on the market, including reproductive and DNT data for those chemicals produced in highest quantity. Chemicals found to be hazardous would be subject to an authorization procedure to show that they can be used safely or that there are no safer alternatives. This registration process would not guarantee that chemicals are safe, but it is a step in the right direction.

The American Chemistry Council has objections to REACH, stating that “the proposed regulation is burdensome, costly, and impractical” (http://www.accnewsmedia.com/site/page.asp?TRACKID=&VID=1&CID=359&DID=1256). The pharmaceutical industry used similar objections to ward off regulations before the thalidomide epidemic ushered in requirements for pharmaceutical agents to undergo extensive premarket testing in clinical trials [80].

## Limitations of Existing Animal Tests for DNT

The US EPA has been slower than the EU to adapt to the overwhelming evidence that low-level exposure to environmental toxins can be harmful. The EPA continues to rely heavily on data from animal (toxicity) testing conducted on only a single animal species and in adult animals. Furthermore, EPA guidelines for a general developmental toxicity screening test typically examine only crude toxicological endpoints such as death, body weight, or organ dysfunction. In contrast, the DNT includes tests of locomotor activity, acoustic startle, learning, and memory. But, as currently designed, the existing tests may miss important effects such as mood changes, impulsive behaviors, and attentional problems that in humans have been shown to result from exposures to environmental toxins [24,27,30,37,40]. While these effects might seem subtle, they can seriously interfere with a child's social and emotional well-being. It is also uncertain whether tests conducted under current EPA guidelines will detect subtle deficits in key human skills such as reading.

There are other problems with relying principally on adult animals to signal the potential for DNT in humans. The structure and development of the cerebral cortex of animals commonly used in these studies differs markedly from that of humans. A chemical's effects on one type of animal may differ from its effects on other animals and on humans. In the case of thalidomide, high-dose fetal exposure had adverse morphologic effects on rabbits, but not rats; functional effects have only recently been described [81].

Although there is some concordance of human and animal data for the adverse effects of lead, mercury, and PCBs, intake limits for these compounds established exclusively on the basis of rodent studies have not been sufficiently protective of human health compared with epidemiologic studies [47]. Indeed, there is compelling evidence from epidemiologic studies of widespread contaminants such as lead, tobacco, and PCBs that human studies are essential to ensure that children are not harmed by low levels of exposure [11,12,13,14,15,16,17,18,19,20,21,22, 23,24,25,26,27,28,29,30,31,32,33,34,35, 36,37,38,39,40].

From a scientific standpoint, data from epidemiologic studies represent the “gold standard” for detecting subtle effects of environmental toxins on humans. But epidemiological studies are expensive to mount, difficult to execute, and take years to complete. Using observational studies to disentangle the adverse consequences of a single toxin from other environmental influences and to promulgate regulations is a difficult and painfully slow process. There is also a financial disincentive for chemical registrants to voluntarily fund such studies because a positive epidemiological study could lead to stricter regulations. More importantly, if society continues to rely on epidemiologic studies to evaluate the toxicity of chemicals only after they are marketed, many children will first be harmed.

## Steps to Protect Children from Environmental Toxins

Children must be better protected from both new and existing chemicals that are known or possible toxins [49]. To protect children from existing toxins, such as lead, mercury, and tobacco, the US EPA and FDA need more authority and resources to regulate and reduce emissions and exposures. Under our current system, efforts to enhance regulations to protect children from confirmed toxins are costly and protracted. Indeed, countless communities across the globe suffer from widespread environmental contamination. If there is any lesson from our experience with environmental toxins, it is that we need to identify environmental chemicals that are toxic before they are marketed or widely disseminated.

For new commercial chemicals, toxicity testing in animals should be required before they are marketed. For all new chemicals, including pesticides, extensive premarket testing should be required in multiple animal species of both sexes and at different developmental stages. These tests should be designed to have adequate statistical power to detect subtle differences within the ranges of exposure that occur in human populations. If implemented, these testing requirements would represent a dramatic departure from existing regulations, while providing a powerful incentive for industry to develop less toxic chemicals.

Toxicity testing in animals is essential but insufficient to protect pregnant women and children. For one thing, uncertainties about the safety of a chemical for humans will persist even after toxicity testing in animals is successfully completed. One additional safeguard that deserves further debate is whether prevalent environmental chemicals to which children could be exposed should undergo more extensive testing in human trials before they are marketed. If done, these trials should examine exposure, uptake (using biomarkers), and adverse effects among children or other populations only when the product is used as intended. For example, once animal toxicity testing of a residential pesticide is complete (including DNT and reproductive toxicity testing), a pesticide could undergo further testing in the home environment. Using an experimental group and a control group, researchers would compare levels of pesticides found in settled dust, on children's hands, and in their blood, urine, or hair. Children would be followed, when indicated, to ensure that an excess of neurobehavioral problems or other relevant outcomes did not develop among those whose homes were assigned to receive the pesticide application.

If such trials were undertaken, families would need to be fully informed about the purpose, potential benefits, and risks of participating. The trials should be conducted by the federal government—or other independent entities that do not have any ties to the chemical industry—and funded by an industry fee or tax. Community representatives would need to be involved in the review and approval of such trials, and ethical standards would need to be established regarding, for example, the role of data safety and monitoring boards. Many families would undoubtedly find it objectionable and would choose not to participate. Indeed, some products might never undergo testing if they failed to offer meaningful benefits to families, in which case the product would either be taken off the market or never reach the market.

This type of trial sounds extreme, but it is quite rational when compared to the existing approach of disseminating a potential toxin into children's environments without any human data about exposure, uptake, or toxicity. Furthermore, under our existing system, families are neither informed nor given an option to decline involvement in what ultimately are experiments exposing millions of pregnant women and children to potential toxins. Thus, we need to thoughtfully deliberate about whether these types of trials can be done in an ethical fashion. We also need to have further debate about whether it is ethical to continue to disseminate chemicals of unknown toxicity into children's environments or to allow children to continually be exposed to prevalent toxins, like lead, despite considerable evidence that they are toxic [82]. Too often, it is left up to a few investigators or community leaders to discover and quantify the adverse effects of toxins, and advocate efforts to reduce children's exposure.

## Conclusion

In contrast with the EU's proposed REACH program, which would require industry to conduct more tests or analyses to demonstrate that high-production chemicals will not cause harm to fetuses or children, the Bush administration has argued—in unison with the American Chemistry Council—that such regulations would harm industry [83,84]. It is time to acknowledge that the existing requirements for toxicity testing and regulations are inadequate to safeguard pregnant women and children. Until a formal regulatory system is developed to effectively screen and identify new and existing chemicals that are toxic to pregnant women and children, we are left to await the next epidemic to warn us about an environmental disaster. Unfortunately, by then we will have once again fouled our nest [85].

**Figure:**
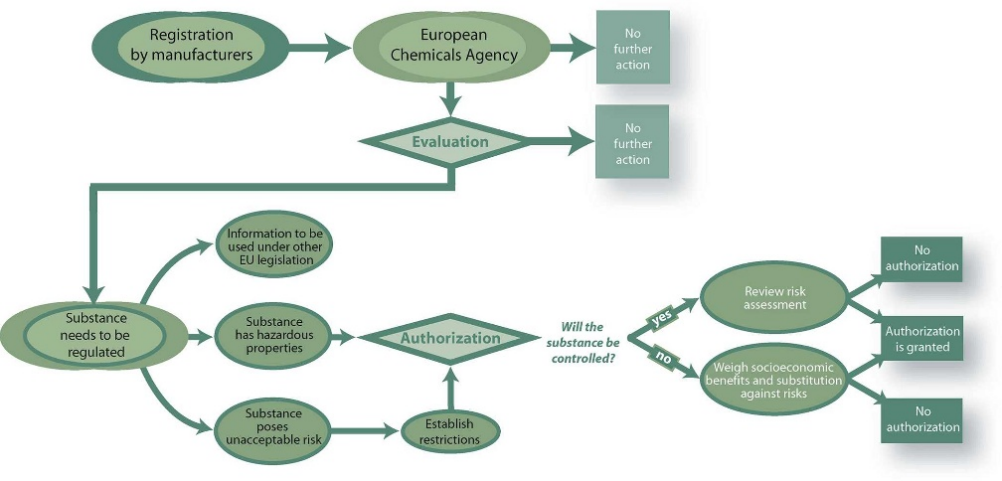
The US framework to protect children from environmental toxins is precarious (Photo: Earl Dotter, http://www.earldotter.com)

## Abbreviations

CBTS: Collaborative Behavioral Teratology Study
DNT: developmental neurotoxicity
EPA: Environmental Protection Agency
FDA: Food and Drug Administration
PCB: polychlorinated biphenyl

## References

[pmed-0020061-b1] Rogan WJ (1995). Environmental poisoning of children: Lessons from the past. Environ Health Perspect.

[pmed-0020061-b2] Gibson JL (1904). A plea for painted railings and painted rooms as the source of lead poisoning amongst Queensland children. Aust Med Gaz.

[pmed-0020061-b3] Turner AJ (1908). Lead poisoning in childhood. Aust Med Congr.

[pmed-0020061-b4] Harada M (1995). Minamata disease: Methylmercury poisoning in Japan caused by environmental pollution. Crit Rev Toxicol.

[pmed-0020061-b5] Chen YC, Guo YL, Hsu CC, Rogan WJ (1992). Cognitive development of Yu-Cheng (“oil disease”) children prenatally exposed to heat-degraded PCBs. JAMA.

[pmed-0020061-b6] Wisborg K, Kesmodel U, Henriksen TB, Olsen SF, Secher NJ (2001). Exposure to tobacco smoke in utero and the risk of stillbirth and death in the first year of life. Am J Epidemiol.

[pmed-0020061-b7] DiFranza JR, Lew RA (1996). Morbidity and mortality associated with use of tobacco products by other people. Pediatrics.

[pmed-0020061-b8] Murray CJ, Lopez AD (1996). Evidence-based health policy: Lessons from the Global Burden of Disease Study. Science.

[pmed-0020061-b9] Ness RB, Grisso JA, Hirschinger N, Markovic N, Shaw LM (1999). Cocaine and tobacco use and the risk of spontaneous abortion. New Engl J Med.

[pmed-0020061-b10] England LJ, Kendrick JS, Wilson HG, Merritt RK, Gargiullo PM (2001). Effects of smoking reduction during pregnancy on the birth weight of term infants. Am J Epidemiol.

[pmed-0020061-b11] National Research Council (2000). Toxicological effects of methylmercury.

[pmed-0020061-b12] Weitzman M, Byrd RS, Aligne CA, Moss M (2002). The effects of tobacco exposure on children's behavioral and cognitive functioning: Implications for clinical and public health policy and future research. Neurotoxicol Teratol.

[pmed-0020061-b13] Windham GC, Hopkins B, Fenster L, Swan SH (2000). Prenatal active or passive tobacco smoke exposure and the risk of preterm delivery or low birth weight. Epidemiology.

[pmed-0020061-b14] Windham GC, Eaton A, Hopkins B (1999). Evidence for an association between environmental tobacco smoke exposure and birthweight: A meta-analysis and new data. Paediatr Perinat Epidemiol.

[pmed-0020061-b15] Torres-Sanchez LE, Berkowitz G, Lopez-Carrillo L, Torres-Arreola L, Rios C (1999). Intrauterine lead exposure and preterm birth. Environ Res.

[pmed-0020061-b16] Needleman HL, Schell A, Bellinger D, Leviton A, Allred EN (1990). The long-term effects of exposure to low doses of lead in childhood. An 11-year follow-up report. N Engl J Med.

[pmed-0020061-b17] Baghurst PA, McMichael AJ, Wigg NR, Vimpani GV, Robertson EF (1992). Environmental exposure to lead and children's intelligence at the age of seven years. The Port Pirie Cohort Study. N Engl J Med.

[pmed-0020061-b18] Bellinger DC, Stiles KM, Needleman HL (1992). Low-level lead exposure, intelligence and academic achievement: A long-term follow-up study. Pediatrics.

[pmed-0020061-b19] Eskenazi B, Trupin LS (1995). Passive and active maternal smoking during pregnancy, as measured by serum cotinine, and postnatal smoke exposure. II. Effects on neurodevelopment at age 5 years. Am J Epidemiol.

[pmed-0020061-b20] Grandjean P, Weihe P, White RF, Debes F, Araki S (1997). Cognitive deficit in 7-year-old children with prenatal exposure to methylmercury. Neurotoxicol Teratol.

[pmed-0020061-b21] Schantz SL, Widholm JJ, Rice DC (2003). Effects of PCB exposure on neuro-psychological function in children. Environ Health Perspect.

[pmed-0020061-b22] Jacobson JL, Jacobson SW (1996). Intellectual impairment in children exposed to polychlorinated biphenyls in utero. N Engl J Med.

[pmed-0020061-b23] Wasserman GA, Liu X, Popovac D, Factor-Litvak P, Kline J (2000). The Yugoslavia Prospective Lead Study: Contributions of prenatal and postnatal lead exposure to early intelligence. Neurotoxicol Teratol.

[pmed-0020061-b24] Dietrich K, Ris M, Succop P, Berger O, Bornshein R (2001). Early exposure to lead and juvenile delinquency. Neurotoxicol Teratol.

[pmed-0020061-b25] Olds DL, Henderson CR, Tatelbaum R (1994). Intellectual impairment in children of women who smoke cigarettes during pregnancy. Pediatrics.

[pmed-0020061-b26] Noland JS, Singer LT, Arendt RE, Minnes S, Short EJ (2003). Executive functioning in preschool-age children prenatally exposed to alcohol, cocaine, and marijuana. Alcohol Clin Exp Res.

[pmed-0020061-b27] Kahn RS, Khoury J, Nichols WC, Lanphear BP (2003). Role of dopamine transporter genotype and maternal prenatal smoking in childhood hyperactive-impulsive, inattentive, and oppositional behaviors. J Pediatr.

[pmed-0020061-b28] Borja-Aburto VH, Hertz-Picciotto I, Rojas Lopez M, Farias P, Rios C (1999). Blood lead levels measured prospectively and risk of spontaneous abortion. Am J Epidemiol.

[pmed-0020061-b29] Jaakkola JJ, Jaakkola N, Zahlsen K (2001). Fetal growth and length of gestation in relation to prenatal exposure to environmental tobacco smoke assessed by hair nicotine concentration. Environ Health Perspect.

[pmed-0020061-b30] Wakschlag LS, Pickett KE, Cook E, Benowitz NL, Leventhal BL (2002). Maternal smoking during pregnancy and severe antisocial behavior in offspring: A review. Am J Public Health.

[pmed-0020061-b31] Needleman HL, Gatsonis CA (1990). Low-level lead exposure and the IQ of children. A meta-analysis of modern studies. JAMA.

[pmed-0020061-b32] Longnecker MP, Klebanoff MA, Zhou H, Brock JW (2001). Association between maternal serum concentration of the DDT metabolite DDE and preterm and small-for-gestational-age babies at birth. Lancet.

[pmed-0020061-b33] Whyatt RM, Rauh V, Barr DB, Camann DE, Andrews HF (2004). Prenatal insecticide exposures and birth weight and length among an urban minority cohort. Environ Health Perspect.

[pmed-0020061-b34] Eskenazi B, Harley K, Bradman A, Weltzien E, Jewell NP (2004). Association of in utero organophosphate pesticide exposure and fetal growth and length of gestation in an agricultural population. Environ Health Perspect.

[pmed-0020061-b35] Berkowitz GS, Wetmur JG, Birman-Deych E, Obel J, Lapinski RH (2004). In utero pesticide exposure, maternal paraoxonase activity, and head circumference. Environ Health Perspect.

[pmed-0020061-b36] Fried PA, Watkinson B, Gray R (1998). Differential effects on cognitive functioning in 9 to 12 year olds prenatally exposed to cigarettes and marijuana. Neurotoxicol Teratol.

[pmed-0020061-b37] Williams GM, O'Callaghan M, Najman JM, Bor W, Andersen MJ (1998). Maternal cigarette smoking and child psychiatric morbidity: A longitudinal study. Pediatrics.

[pmed-0020061-b38] Chiodo LM, Jacobson SW, Jacobson JL (2004). Neurodevelopmental effects of postnatal lead exposure at very low levels. Neurotoxicol Teratol.

[pmed-0020061-b39] Stewart PW, Reihman J, Lonky EI, Darvill TJ, Pagano J (2003). Cognitive development in preschool children prenatally exposed to PCBs and MeHg. Neurotoxicol Teratol.

[pmed-0020061-b40] Sood B, Delaney-Black V, Covington C, Nordstrom-Klee B, Ager J (2001). Prenatal alcohol exposure and childhood behavior at age 6 to 7 years: I dose-response effect. Pediatrics.

[pmed-0020061-b41] Lanphear BP, Dietrich KN, Auinger P, Cox C (2000). Cognitive deficits associated with blood lead levels < 10 µg/dl in U.S. children and adolescents. Public Health Rep.

[pmed-0020061-b42] Canfield RL, Henderson CR, Cory-Slechta DA, Cox C, Jusko TA (2003). Intellectual impairment in children with blood lead concentrations below 10 micrograms per deciliter. N Engl J Med.

[pmed-0020061-b43] Yolton K, Auinger P, Dietrich KN, Lanphear BP, Hornung R (2005). Exposure to environmental tobacco smoke and cognitive abilities among US children and adolescents. Environ Health Perspect.

[pmed-0020061-b44] National Research Council (1993). Pesticides in the diets of infants and children.

[pmed-0020061-b45] Centers for Disease Control and Prevention (2003). Second national report on human exposure to environmental chemicals.

[pmed-0020061-b46] Rice D, Barone S (2000). Critical periods of vulnerability for the developing nervous system: Evidence from humans and animal models. Environ Health Perspect.

[pmed-0020061-b47] Rice DC, Evangelista de Duffard AM, Duffard R, Iregren A (1996). Lessons for neurotoxicology from selected model compounds: SGOMSEC joint report. Environ Health Perspect.

[pmed-0020061-b48] Weiss B, Spyker JM (1974). The susceptibility of the fetus and child to chemical pollutants. Behavioral implications of prenatal and early postnatal exposure to chemical pollutants. Pediatrics.

[pmed-0020061-b49] Claudio L, Kwa WC, Russell AL, Wallinga D (2000). Testing methods for developmental neurotoxicity of environmental chemicals. Toxicol Appl Pharmacol.

[pmed-0020061-b50] Claudio L, Bearer CF, Wallinga D (1999). Assessment of the U.S. Environmental Protection Agency methods for identification of hazards to developing organisms, Part II: The developmental toxicity testing guideline. Am J Ind Med.

[pmed-0020061-b51] Jacobson JL, Jacobson SW (1997). Teratogen update: Polychlorinated biphenyls. Teratology.

[pmed-0020061-b52] Lanphear BP, Hornung R, Ho M, Howard CR, Eberly S (2002). Environmental lead exposure during early childhood. J Pediatr.

[pmed-0020061-b53] Noren K, Meironyte D (1998). Contaminants in Swedish human milk: Decreasing levels of organochlorines and increasing levels of organobromine compounds. Organohalogen Compounds.

[pmed-0020061-b54] Curl CL, Fenske RA, Elgethun K (2003). Organophosphorus pesticide exposure of urban and suburban preschool children with organic and conventional diets. Environ Health Perspect.

[pmed-0020061-b55] Ramirez GB, Cruz MC, Pagulayan O, Ostrea S, Dalisay C (2000). The Tagum study I: Analysis and clinical correlates of mercury in maternal and cord blood, breast milk, meconium, and infants' hair. Pediatrics.

[pmed-0020061-b56] Whyatt RM, Jedrychowski W, Hemminki K, Santella RM, Tsai WY (2001). Biomarkers of polycyclic aromatic hydrocarbon-DNA damage and cigarette smoke exposures in paired maternal and newborn blood samples as a measure of differential susceptibility. Cancer Epidemiol Biomarkers Prev.

[pmed-0020061-b57] Perera FP (1997). Environment and cancer: Who are susceptible?. Science.

[pmed-0020061-b58] Boyle CA, Decoufle P, Yeargin-Allsopp M (1994). Prevalence and health impact of developmental disabilities in US children. Pediatrics.

[pmed-0020061-b59] Sampson PD, Streissguth AP, Bookstein FL, Little RE, Clarren SK (1997). Incidence of fetal alcohol syndrome and prevalence of alcohol-related neurodevelopmental disorder. Teratology.

[pmed-0020061-b60] Drews CD, Murphy CC, Yeargin-Allsopp M, Decoufle P (1996). The relationship between idiopathic mental retardation and maternal smoking during pregnancy. Pediatrics.

[pmed-0020061-b61] Schettler T (2001). Toxic threats to neurologic development of children. Environ Health Perspect.

[pmed-0020061-b62] Branum AM, Schoendorf KC (2002). Changing patterns of low birthweight and preterm birth in the United States, 1981–1998. Paediatr Perinat Epidemiol.

[pmed-0020061-b63] Demissie K, Rhoads GG, Ananth CV, Alexander GR, Kramer MS (2001). Trends in preterm birth and neonatal mortality among blacks and whites in the United States from 1989 to 1997. Am J Epidemiol.

[pmed-0020061-b64] Hack M, Flannery DJ, Schluchter M, Cartar L, Borawski E (2002). Outcomes in young adulthood for very-low-birth-weight infants. N Engl J Med.

[pmed-0020061-b65] Korrick SA, Chen C, Damokosh AI, Ni J, Liu X (2001). Association of DDT with spontaneous abortion: A case-control study. Ann Epidemiol.

[pmed-0020061-b66] Kharrazi M, DeLorenze GN, Kaufman FL, Eskenazi B, Bernert JT (2004). Environmental tobacco smoke and pregnancy outcome. Epidemiology.

[pmed-0020061-b67] Department of Developmental Services (1999). Changes in the population of persons with autism and pervasive developmental disorders in California's developmental services system: 1987 through 1998. Sacramento (California): Department of Developmental Services. http://www.dds.ca.gov/Autism/pdf/Autism_Report_1999.PDF.

[pmed-0020061-b68] Yeargin-Allsopp M, Rice C, Karapurkar T, Doernberg N, Boyle C (2003). Prevalence of autism in a US metropolitan area. JAMA.

[pmed-0020061-b69] Zito JM, Safer DJ, dosReis S, Gardner JF, Boles M (2000). Trends in the prescribing of psychotropic medications to preschoolers. JAMA.

[pmed-0020061-b70] Akinbami LJ, Schoendorf KC (2002). Trends in childhood asthma: Prevalence, health care utilization, and mortality. Pediatrics.

[pmed-0020061-b71] Wynder E (1994). Invited commentary: Studies in mechanism and prevention. Striking a proper balance. Am J Epidemiol.

[pmed-0020061-b72] Miller MT, Stromland K (1999). Teratogen update. Thalidomide: A review, with a focus on ocular findings and new potential uses. Teratology.

[pmed-0020061-b73] Vorhees CV, Riley EP, Vorhees CV (1986). Comparison and critique of government regulations for behavioral teratology. Handbook of behavioral teratology.

[pmed-0020061-b74] Kimmel CA, Buelke-Sam J (1985). Collaborative Behavioral Teratology Study: Background and overview. Neurobehav Toxicol Teratol.

[pmed-0020061-b75] Makris S, Raffaele K, Sette W, Seed J (1998). A retrospective analysis of twelve developmental neurotoxicity studies submitted to the USEPA Office of Prevention, Pesticides and Toxic Substances (OPPTS). http://www.epa.gov/scipoly/sap/1998/december/neuro.pdf.

[pmed-0020061-b76] Goldman LR (1998). Linking research and policy to ensure children's environmental health. Environ Health Perspect.

[pmed-0020061-b77] Stein J, Schettler T, Wallinga D, Valenti M (2002). In harm's way: Toxic threats to children's development. J Dev Behav Pediatr.

[pmed-0020061-b78] European Commission (2004). REACH: The strategy for a future chemicals policy. http://europa.eu.int/comm/enterprise/reach/whitepaper/index.htm.

[pmed-0020061-b79] Brown VJ (2003). REACHing for chemical safety. Environ Health Perspect.

[pmed-0020061-b80] Hilts P (2003). Protecting America's health: The FDA, business, and one hundred years of regulation.

[pmed-0020061-b81] Vorhees CV, Weisenberger WP, Minck DR (2001). Neurobehavioral teratogenic effects of thalidomide in rats. Neurotoxicol Teratol.

[pmed-0020061-b82] Lanphear BP (1998). The paradox of lead poisoning prevention. Science.

[pmed-0020061-b83] Loewenberg S (2003 March 18). Precaution is for Europeans. The New York Times.

[pmed-0020061-b84] Becker E (2004 April 2). White House undermined chemical tests, report says. The New York Times.

[pmed-0020061-b85] Chisolm JJ (1978). Fouling one's own nest. Pediatrics.

[pmed-0020061-b86] Commission of the European Communities (2004). Flowcharts on the new EU chemicals legislation REACH. http://europa.eu.int/comm/enterprise/reach/docs/reach/flowchart-2004_04_04.pdf.

